# Genome-wide identification and characterization of the *bZIP* gene family and their function in starch accumulation in Chinese chestnut (*Castanea mollissima* Blume)

**DOI:** 10.3389/fpls.2023.1166717

**Published:** 2023-04-03

**Authors:** Penglong Zhang, Jing Liu, Nan Jia, Meng Wang, Yi Lu, Dongsheng Wang, Jingzheng Zhang, Haie Zhang, Xuan Wang

**Affiliations:** ^1^ Engineering Research Center of Chestnut Industry Technology, Ministry of Education, Qinhuangdao, Hebei, China; ^2^ Hebei Key Laboratory of Horticultural Germplasm Excavation and Innovative Utilization, College of Horticulture Science and Technology, Hebei Normal University of Science and Technology, Changli, Hebei, China; ^3^ Changli Institute of Pomology, Hebei Academy of Agriculture and Forestry Science, Changli, Hebei, China

**Keywords:** *Castanea mollissima*, bZIP, gene family, starch accumulation, yeast one-hybrid, CmbZIP13, CmbZIP35

## Abstract

The transcription factors of basic leucine zipper (bZIP) family genes play significant roles in stress response as well as growth and development in plants. However, little is known about the *bZIP* gene family in Chinese chestnut (*Castanea mollissima* Blume). To better understand the characteristics of *bZIP*s in chestnut and their function in starch accumulation, a series of analyses were performed including phylogenetic, synteny, co-expression and yeast one-hybrid analyses. Totally, we identified 59 *bZIP* genes that were unevenly distributed in the chestnut genome and named them *CmbZIP01* to *CmbZIP59*. These *CmbZIP*s were clustered into 13 clades with clade-specific motifs and structures. A synteny analysis revealed that segmental duplication was the major driving force of expansion of the *CmbZIP* gene family. A total of 41 *CmbZIP* genes had syntenic relationships with four other species. The results from the co-expression analyses indicated that seven *CmbZIP*s in three key modules may be important in regulating starch accumulation in chestnut seeds. Yeast one-hybrid assays showed that transcription factors CmbZIP13 and CmbZIP35 might participate in starch accumulation in the chestnut seed by binding to the promoters of *CmISA2* and *CmSBE1_2*, respectively. Our study provided basic information on *CmbZIP* genes, which can be utilized in future functional analysis and breeding studies

## 1 Introduction

Starch is an important form of carbon storage for the majority of plant species. Throughout the lifecycle of a plant, starch plays roles in development and response to the environment (MacNeill et al., 2017). Short-term storage of starch occurs in the leaves of plants, whereas long-term storage takes place in seeds and tubers, providing material for energy, development, and reproduction (MacNeill et al., 2017). A previous study has reported that starch can be degraded by a kind of α-amylase in response to osmotic stress (Thalmann et al., 2016). In many plants, the starch biosynthesis pathway is catalyzed by enzymes, such as ADP-glucose pyrophosphorylase, starch synthase, starch branching enzyme, and starch de-branching enzyme (Qu et al., 2018), and regulated by transcription factors (TFs) (MacNeill et al., 2017). For example, as an AP2/EREBP TF family member, rice starch regulator 1 negatively regulates the expression of starch synthesis-related genes in rice seeds and is involved in the amylose content of the seed (Fu and Xue, 2010). The endosperm-specific TF TaNAC019 can bind to the promoters of starch metabolism genes, regulate starch accumulation, and improve the quality of wheat grains (Gao et al., 2021).

TFs play an indispensable role in plant growth, development, and resistance to biotic and abiotic stresses (Wang et al., 2022). As one of the largest and most diverse TF families, the basic leucine zipper (bZIP) family has been studied extensively (Lee et al., 2006; Schlögl et al., 2008; Wang et al., 2013; An et al., 2018; Song et al., 2020; Duan et al., 2022). The members of the bZIP TF family harbor a highly conserved, 60–80 amino acid long domain, which is composed of a basic region and a leucine zipper region. The sequence of the basic region consists of approximately 20 amino acid residues with a fixed nuclear localization structure N-x7-R/K, which can specifically bind to DNA cis-elements (Lee et al., 2006; Nijhawan et al., 2008). The leucine zipper region, containing the core sequences of L-x6-L-x6-L, consists of various repetitions of leucine or other hydrophobic amino acids, which facilitates hetero- or homo-dimerization of bZIP proteins (Jakoby et al., 2002). The core sequence recognized by bZIP TFs is ACGT, which includes an A-box (TACGTA), C-box (GACGTC), and G-box (CACGTG) (Izawa et al., 1993). Most abscisic acid induced genes have these ACGT cis-elements in their promoter regions. In addition, bZIP TFs can also recognize non-palindrome sequences, such as H-box (CCTACC), GCN4-like motif (GTGAGTCAT), and prolamin box-like (TGAAAA) elements (Kim et al., 2014).

The bZIP TF genes play an important role in many plant biological processes, such as regulating plant morphology and growth. For example, over-expression of the pepper *CabZIP1* gene in *Arabidopsis* slowed plant growth and reduced the number of petals (Lee et al., 2006). The bZIP TFs of tobacco regulate its transition from vegetative growth to reproductive growth (Heinekamp et al., 2002). Plant bZIP transcription factors are induced by plant hormones such as salicylic acid, methyl jasmonate, ethylene, or abscisic acid (Meng et al., 2005; Lee et al., 2006; Schlögl et al., 2008). In addition, many bZIP transcription factors can regulate abscisic acid synthesis, which regulates gene expression (Finkelstein and Lynch, 2000; Uno et al., 2000). bZIP family genes participate in the regulation of plant resistance to biotic and/or abiotic stresses. Overexpression of bZIP-like proteins in plants under stress conditions can improve the photosynthetic capacity of plants, improving their resistance to salt, cold, herbicides, drought, heat, and other stresses (Kim et al., 2004; Lee et al., 2006; Liao et al., 2008; Zhang et al., 2008). For example, the silencing of the rice endogenous *rT-GA2.1* gene (a member of the bZIP family) mediated by dsRNA can improve the resistance of rice to bacterial pathogens, such as *Xanthomonas oryzae* pv. oryzae, indicating that *rTGA2.1* plays a negative role in response to bacterial pathogens (Fitzgerald et al., 2005). Some members of the bZIP family also act as regulators in the starch synthesis pathway. In rice, OsbZIP20, OsbZIP33, and OsbZIP58 TFs interact with *granule-bound starch synthase* (*GBSS*) and *starch branching enzyme 1* (*SBE1*) genes by binding to their promoters, and are capable of regulating starch synthesis (Cai et al., 2002; Wang et al., 2013). In wheat, *TubZIP28* (from *Triticum urartu*) and *TabZIP28* (from *Triticum aestivum*) also participate in the regulation of starch synthesis by interacting with starch synthesis-related genes (Song et al., 2020)

Many bZIP TF families have been identified in important plant species. For example, 75 *bZIP* genes have been predicted in *Arabidopsis thaliana* (Jakoby et al., 2002). One hundred twenty-five members of the *bZIP* genes family were identified in maize (*Zea mays*) (Wei et al., 2012), 114 in apple (*Malus domestica*) (Li et al., 2016), 92 in pear (*Pyrus breschneideri*) (Ma et al., 2021), 77 in tobacco (*Nicotiana tabacum*) (Duan et al., 2022), 65 in pomegranate (*Punica granatum*) (Wang et al., 2022), and 227 in wheat (*Triticum aestivum*) (Liang et al., 2022). However, there are no reports on the identification and functionality of *bZIP* genes in Chinese chestnut (*Castanea mollissima*), despite Chinese chestnut being an economically important dry fruit tree species that is favorited for its sweet, fragrant, and waxy characteristics. The waxiness is a critical parameter for chestnut quality and is determined by the starch content in the kernel (Lin et al., 2012; Shi et al., 2021). Over the past four years, several versions of the *C. mollissima* genome have been assembled and published (Xing et al., 2019; Sun et al., 2020; Wang et al., 2020; Hu et al., 2022). These assemblies are resources for the identification of gene families and genetic improvement of chestnut.

In this study, we aimed to identify *bZIP* genes from Chinese chestnut using the whole genome of N11-1, a seedling Chinese chestnut cultivar line (Wang et al., 2020). We performed phylogenetic, conserved motif, and gene structure analyses to study the relationships between the identified *CmbZIP* family members. Whole genome duplication (WGD) analysis revealed that segmental duplication might be the main factor that led to the expansion of the *CmbZIP* family. Transcriptomic data from different development stages of chestnut seeds showed that *CmbZIP* genes had different expression patterns, and some of them may be related to starch accumulation. These results from this study provide a theoretical reference for *CmbZIP* genes and insight useful for future studies on Chinese chestnut.

## 2 Results

### 2.1 Identification and characterization of *CmbZIP* genes

In this study, we identified 59 *bZIP* gene family members from the whole genome of the seedling Chinese chestnut cultivar N11-1, which version provided relatively complete annotative information at the chromosome level (Wang et al., 2020). For subsequent analysis, we named these genes *CmbZIP01* to *CmbZIP59* based on the chromosome and/or contig location (**Table S1**). The molecular weight of chestnut bZIP family proteins ranged from 16,067.34 to 122,096.53 Da, the theoretical isoelectric point ranged from 4.62 to 9.76, and the protein length ranged from 140 (CmbZIP52) to 1,075 aa (CmbZIP28). Fifty-seven CmbZIP proteins were located in the nuclear region, whereas CmbZIP35 and CmbZIP41 were located in the chloroplast and vacuole, respectively (**Table S1**). These results provide a theoretical basis for further purification, activity, and functional studies of CmbZIP proteins.

### 2.2 Phylogenetic analysis and classification of the chestnut bZIP TF family

**Figure 1:**
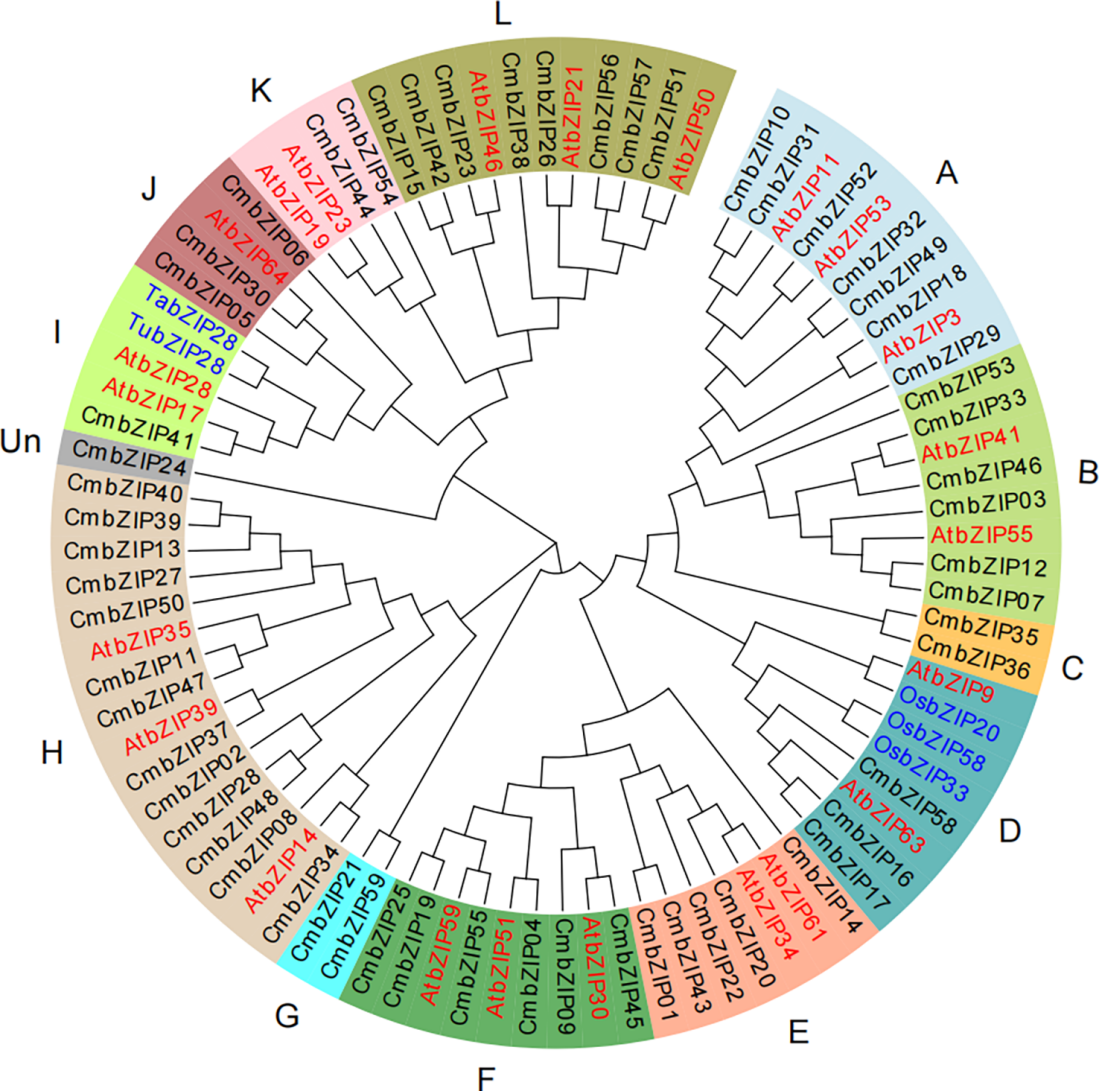
The phylogenetic tree of bZIP proteins in chestnut, *Arabidopsis*, rice, and wheat. The proteins are clustered in 13 clades (A–L, Un) by pre-grouping with AtbZIPs (Jakoby et al., 2002). Different background colors indicate different bZIP protein clades. The black, red, and blue font indicate the bZIP proteins of chestnut, *Arabidopsis*, and rice and wheat, respectively.

To explore the homologous evolutionary relationships and classification of the bZIP family, we constructed an unrooted neighbor-joining phylogenetic tree using bZIP protein sequences from chestnut, *Arabidopsis*, and five other bZIP proteins reported in previous studies: OsbZIP20 (Izawa et al., 1994), OsbZIP33 (Nakase et al., 1997), OsbZIP58 (Wang et al., 2013), TubZIP28, and TabZIP28 (Song et al., 2020). The 59 CmbZIP proteins were divided into 13 clades (A, B, C, D, E, F, G, H, I, J, K, L, and Un) according to their homology in *Arabidopsis* (Jakoby et al., 2002) (**Figure 1**). The number of CmbZIP proteins in the 13 clades differed greatly in size. The largest clade (H) had 13 members. CmbZIP24 was clustered into the smallest, unique clade (Un) in the phylogenetic tree and might have an evolutionary trajectory unrelated to other clades. Two of the clades had only one CmbZIP TF member: clade I and clade Un (**Figure 1**; **Table S1**). The bZIP proteins of the four species included in this analysis were separately distributed throughout the 13 clades in the phylogenetic tree, indicating that the bZIP proteins showed similar divergences in gene function in chestnut, *Arabidopsis*, rice, and wheat. Some bZIP proteins clustered together in a small clade, suggesting that a co-speciation event and species-specific duplication events occurred during the divergence of the bZIP TF family. Our analysis revealed that three homologous proteins, CmbZIP16, CmbZIP17, and CmbZIP58, in clade D and CmbZIP41 in clade I, were able to influence starch accumulation, which is similar to the evolutionary relationships in *Arabidopsis*.

### 2.3 Conserved motif and structure analyses of chestnut *bZIP* genes

**Figure 2:**
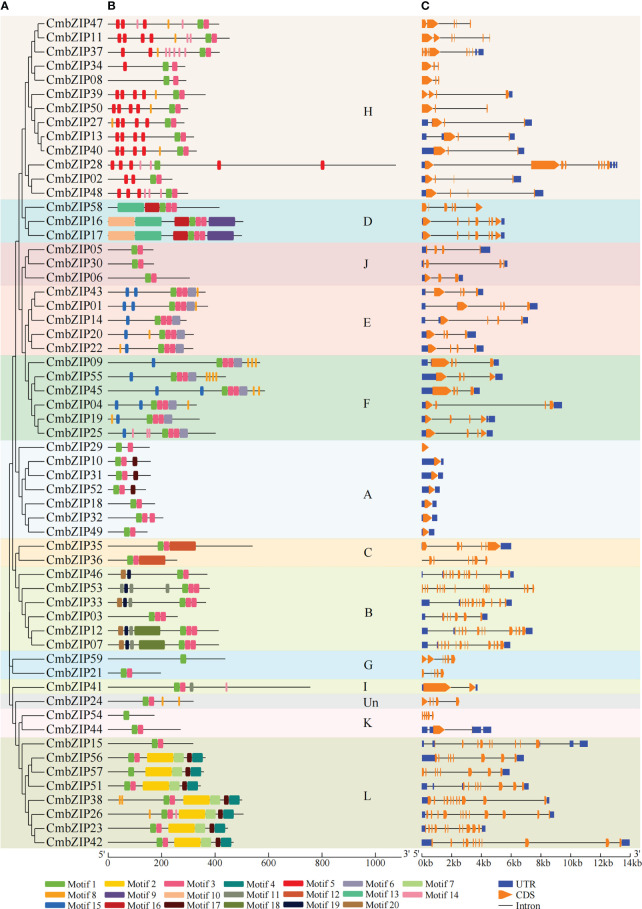
Conserved motif patterns and structure schematics of CmbZIPs. **(A)** The phylogenetic tree was derived from 59 CmbZIP proteins. **(B)** Conserved motif analysis of CmbZIP proteins with 20 separate patterns depicted with different colors. **(C)** Analysis of *CmbZIP* gene structure. The clade names A–L and Un are the same as in **Figure 1**.

In order to study the characteristics of the 59 CmbZIP proteins, we identified 20 conserved motifs varying from eight (motif 8 and 14) to 100 (motif 2, 9, 10, 12 and 13) aa residues long (**Figure 2**; **Table S2**). Motifs 1 and 3 were widely distributed in tandem in almost all (55 of 59) CmbZIP proteins; further sequence analysis indicated that these two motifs constitute the DNA binding basic region and the leucine zipper region of the bZIP domain, respectively (**Figure S1**). In addition, the distribution of 16 motifs showed clade specificity in the phylogenetic tree presented in **Figure 2** (**Table S3**). Motifs 2, 4, 7, and 17 formed two dimers (motifs 2–7 and motifs 17–4), which were present in seven members of clade L. Motif 17 was identified in clade A. Similarly, motif 11 was present in both clades B and I. Motif 5 was only distributed in clade H, except for CmbZIP08, which contained this motif one to five times. Furthermore, motifs 6 and 15 that had a similar pattern were predicted in all members of clades E and F; these two clades were sister to each other in the phylogenetic tree (**Figure 2**). Eight other motifs were specifically distributed in clade B (motifs 18, 19, and 20), C (motif 12), and D (motifs 9, 10, 13, and 16) (**Figure 2**).

For further insight into the evolution of *bZIP* genes in chestnut, we compared the DNA sequences and examined the organization of exons and introns in open reading frames of *CmbZIP* genes. In total, the number of introns in the *CmbZIP*s ranged from 0 to 13. Fifteen *CmbZIP*s contained three introns, accounting for the largest proportion of identified *bZIP* genes (25.4%). *CmbZIP15* (with eight introns) and *CmbZIP53* (with 13 introns), respectively, accounted for the smallest proportion of identified *bZIP* genes (1.6%) (**Table S1**). As expected, members of the same clade had relatively conservative numbers of introns. Seven *CmbZIP*s, all members of clade A, did not have any introns. All *bZIP* genes in clade F contained three introns. Furthermore, the number of introns in clade B varied from five to 13, two to seven in H, and seven to 11 in clade L (**Figure 2C**; **Table S1**). Overall, similar exon–intron structure and motif composition were observed for *bZIP* genes of chestnut in the same clade; the structure and motif composition differ between clades, illustrating that the evolution and divergence of *CmbZIP*s might have occurred at an early stage of the evolution of *C. mollisima*.

### 2.4 Chromosome location and duplication of *bZIP*s in chestnut

**Figure 3:**
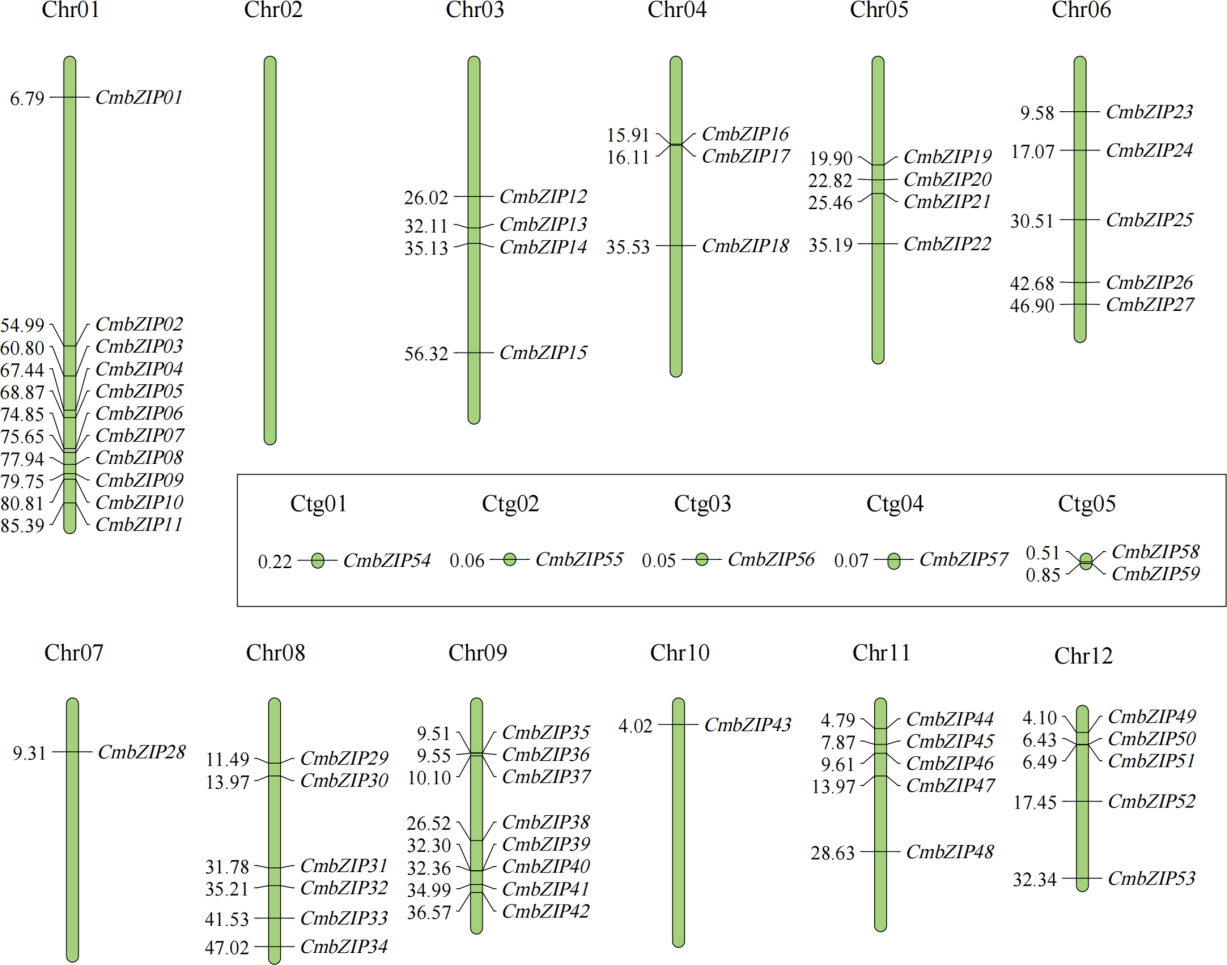
Chromosome locations of *CmbZIP* genes. Vertical green bars represent the chromosomes of chestnut. The chromosome number is stated at the top of each chromosome. The numbers on the left of vertical bars indicate the corresponding physical positions of *CmbZIP*s. The locations of *CmbZIP*s on the contigs are shown in the rectangle.

Fifty-nine *CmbZIP* genes were distributed unevenly on 11 of the 12 chromosomes (Chr) of chestnut as well as five contigs (Ctg) (**Figure 3**; **Table S1**). Chr02 did not contain any *CmbZIP* genes. There was only one *bZIP* gene on the following chromosomes: Chr07 (*CmbZIP28*), Chr10 (*CmbZIP43*), Ctg1 (*CmbZIP54*), Ctg2 (*CmbZIP55*), Ctg3 (*CmbZIP56*), and Ctg4 (*CmbZIP57*); there were two *bZIP* genes on Ctg5 (*CmbZIP58* and *CmbZIP59*). Eleven *CmbZIP* genes (18.64%) were located on Chr01, which contained the greatest number of *bZIP* genes, with 1 and 10 *CmbZIP*s, respectively, located on the proximate and distal ends of this chromosome. Five *CmbZIP*s were relatively evenly dispersed throughout Chr06. Three to eight *bZIP* genes were located on the remaining eight chromosomes.

**Figure 4:**
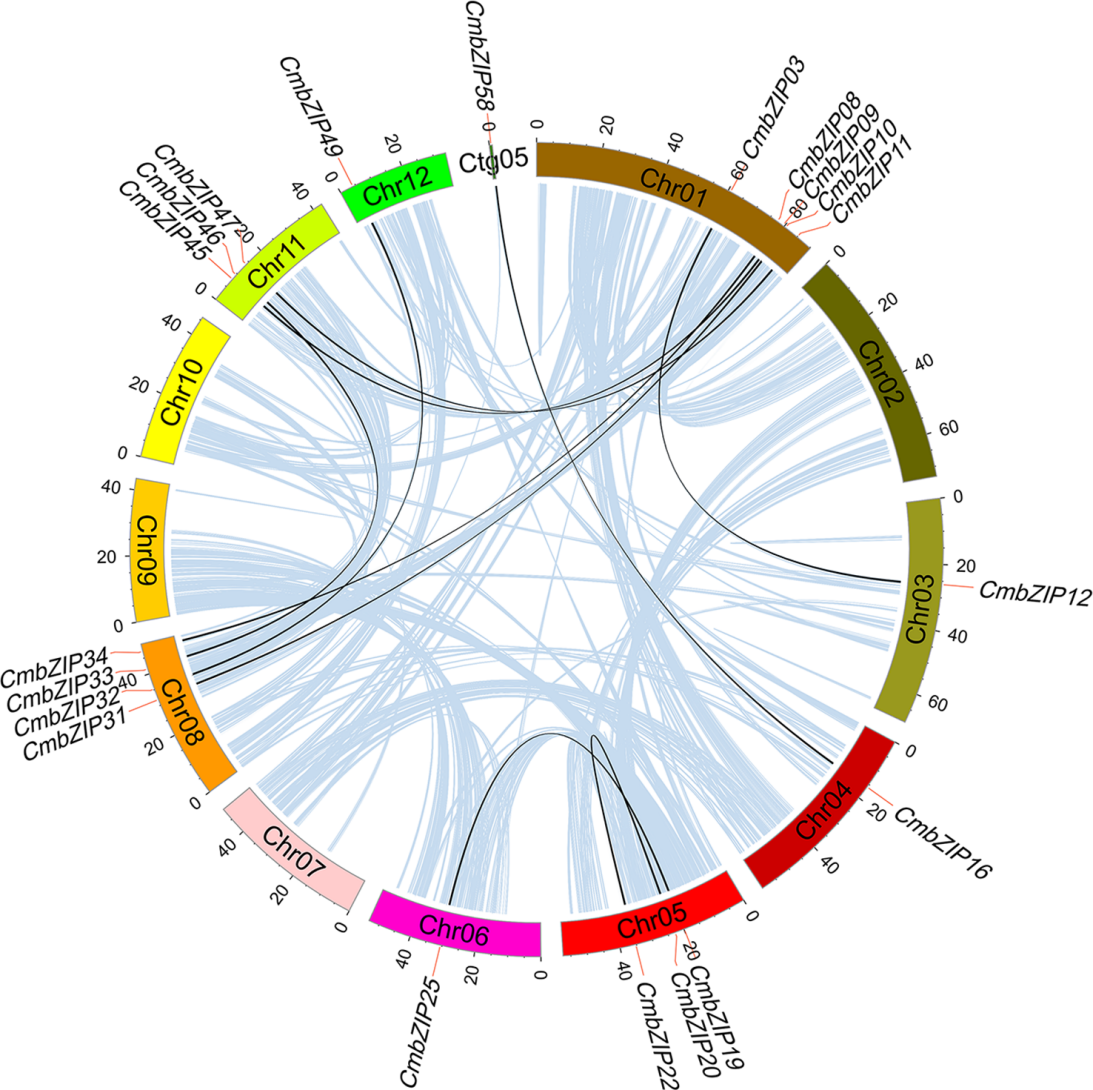
The syntenic pairs of *CmbZIP* genes from different modes of duplication events. Colored rectangles represent chromosome, and the chromosome number is inside these rectangles. Light blue lines indicate all synteny blocks in the chestnut genome; black lines indicate duplicated *bZIP* gene pairs. Names of *bZIP* genes are marked at the position corresponding to the red lines.

We detected 10 pairs of segmental duplications in *CmbZIP* genes, and no tandem duplications were observed, indicating that segmental duplication events were the major cause of expansion of the *CmbZIP* family (**Figure 4**; **Table 1**). Chromosomal distribution analysis revealed that the 20 analogous *CmbZIP*s were unevenly located on the Chinese chestnut genome. We also found that every pair of duplicated *CmbZIP*s were in clades A, B, D, E, F, and H (**Table 1**). Furthermore, we calculated the synonymous substitution rate (Ks) to estimate the segmental duplication events for *CmbZIP*s (**Table 1**). The divergence time of all duplicated *CmbZIP*s varied greatly from 9.50–116.09 million years ago (Mya). To predict the selection pressure driving the divergence of *CmbZIP*s, we also calculated the nonsynonymous substitution rate (Ka) and the Ka/Ks ratio. Seven pairs of duplicated *CmbZIP*s might have undergone purifying selection from 27.74–116.09 Mya. The selection pressure on *CmbZIP20*/*22* was the strongest (Ka/Ks=0.06). Conversely, *CmbZIP09/45* and *CmbZIP19/25* might have recently undergone positive selection from 9.50–11.65 Mya.

**Table 1:** Estimation of the date of segmental duplication events for *CmbZIP*s.

Duplicated gene pairs		Clade		Ka	Ks	Ka/Ks	Divergence time (Mya)
gene 1	gene 2	gene 1	gene 2				
*CmbZIP20*	*CmbZIP22*	E	E	0.22	3.48	0.06	116.09
*CmbZIP03*	*CmbZIP12*	B	B	0.55	2.42	0.23	80.65
*CmbZIP08*	*CmbZIP34*	H	H	0.39	2.29	0.17	76.43
*CmbZIP49*	*CmbZIP32*	A	A	0.55	1.60	0.35	53.17
*CmbZIP46*	*CmbZIP33*	B	B	0.48	1.43	0.34	47.79
*CmbZIP10*	*CmbZIP31*	A	A	0.21	1.35	0.15	45.01
*CmbZIP11*	*CmbZIP47*	H	H	0.47	0.83	0.56	27.74
*CmbZIP58*	*CmbZIP16*	D	D	1.01	0.96	1.05	32.08
*CmbZIP09*	*CmbZIP45*	F	F	0.57	0.35	1.63	11.65
*CmbZIP19*	*CmbZIP25*	F	F	0.47	0.29	1.64	9.50

*Ka, the nonsynonymous substitution rate; Ks, the synonymous substitution rate; Mya, million years ago.

### 2.5 Synteny analysis of *bZIP*s between genomes

**Figure 5:**
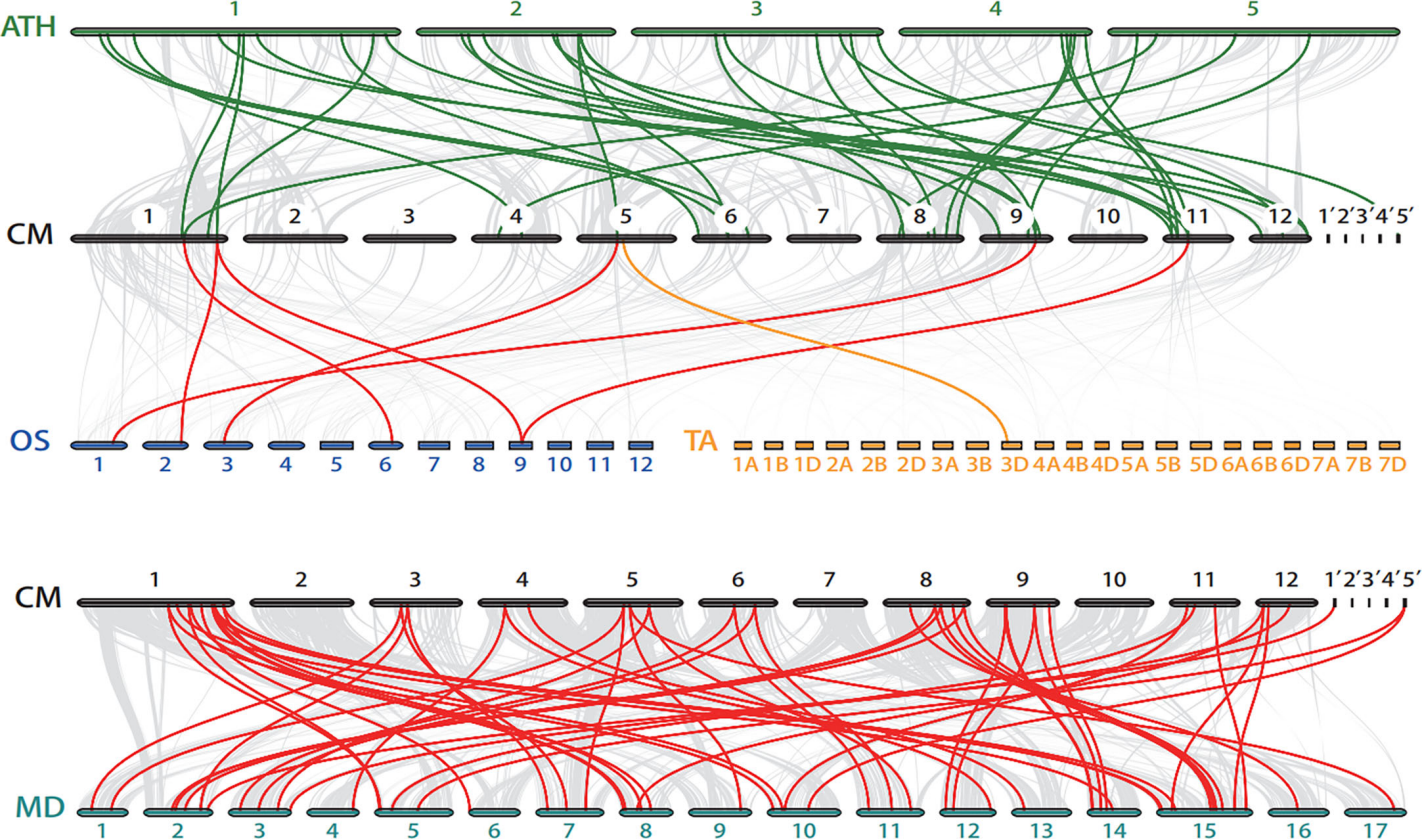
Synteny analysis of the *bZIP* genes between chestnut and four other plant species. The grey lines in the background indicate the collinear blocks between the chestnut genome and other genomes. Bold, colored lines highlight the syntenic *bZIP* gene pairs. The colored bars represent chromosomes of different species. 1’-5’: Ctg1-Ctg5, contigs in chestnut genome. CM, *Castanea mollissima*; ATH, *Arabidopsis thaliana*; OS, *Oryza sativa*; TA, *Triticum aestivum*; MD, *Malus domestica*.

To gain deeper insight into the evolutionary relationships of the *bZIP* genes family among different species, we constructed four comparative syntenic maps of chestnut associated with *Arabidopsis*, rice, wheat, and apple (**Figure 5**; **Table S4**). In total, there were 41 orthologous *bZIP* gene pairs between chestnut and the other four species. Further, we found 31 *CmbZIP*s associated with at least two syntenic gene pairs, suggesting that these genes might play an important role in the evolutionary process of the *bZIP* family. Thirty-four *CmbZIP*s showed syntenic relationships with apple *bZIP* genes, 27 with *Arabidopsis*, five with rice, and one with wheat. There was a far greater number of syntenic *bZIP* pairs between chestnut and the two dicots (i.e., apple and *Arabidopsis*) than between chestnut and the two monocots (i.e., rice and wheat), which might indicate that most of these orthologous pairs occurred after the divergence of dicotyledons and monocotyledons.

### 2.6 Analysis of expression patterns and identification of *CmbZIP*s related to starch accumulation

**Figure 6:**
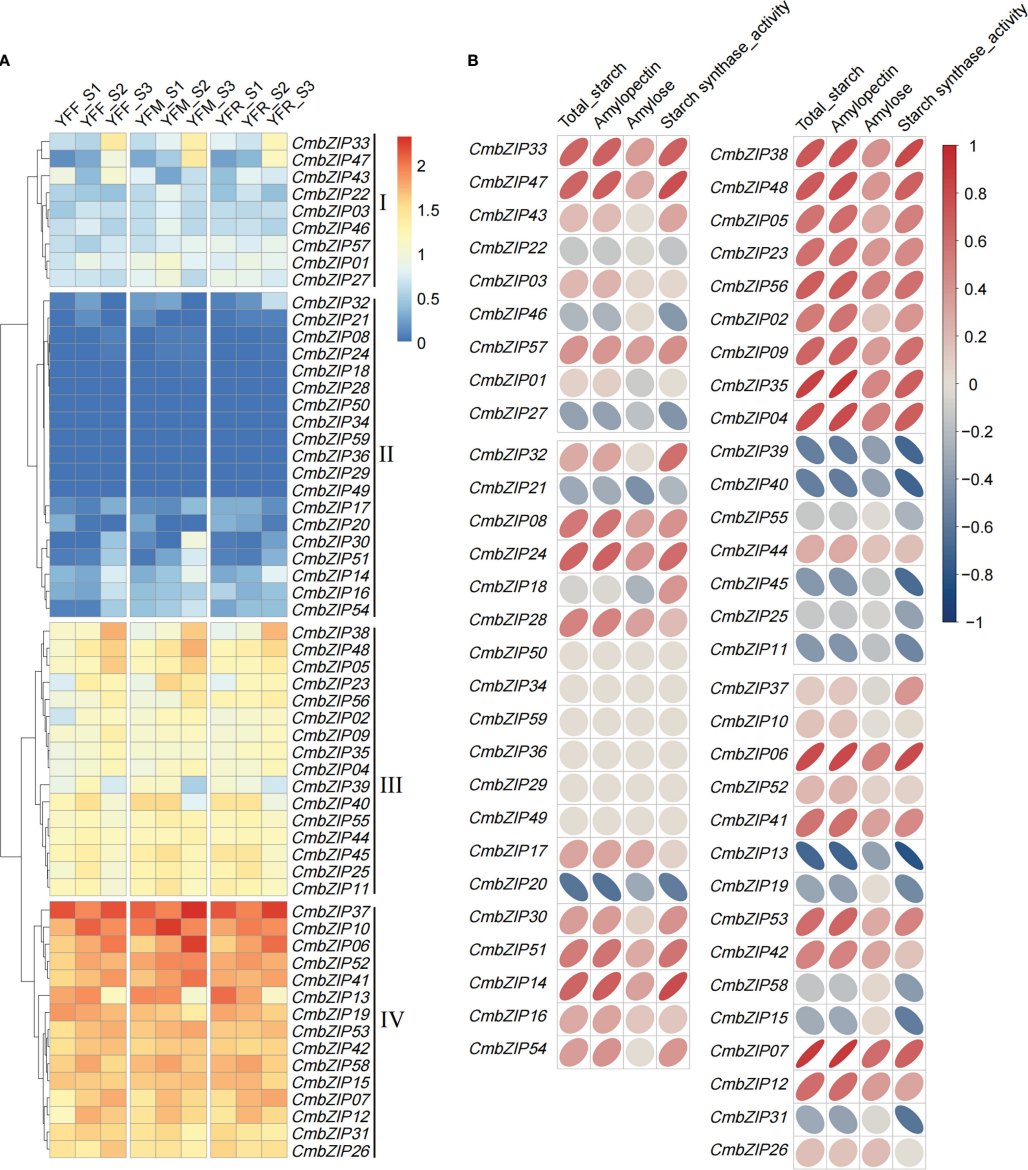
Expression pattern of 59 *CmbZIP* genes and correlation analysis of *CmbZIP* expression and physiological characteristics of three chestnut crosses (Li et al., 2021). **(A)** The heatmap shows the expression pattern of *CmbZIP* genes. YFF, YFM, and YFR indicate seeds from crosses of ‘Yongfeng 1’×’Yongfeng 1,’ ‘Yongfeng 1’×’Yimen 1,’ and ‘Yongfeng 1’×’Yongren Zao,’ respectively. S1, S2, and S3 indicate 70, 82, and 94 days after pollination, respectively. The Roman numerals along the right-hand side of the figure indicate log_10_ FPKM. **(B)** The correlation coefficients between the expression of *CmbZIP*s and four traits are shown by elliptical bubbles. The flatter the bubble, the higher the correlation. Dark red (from top right to bottom left) and dark blue (from top left to bottom right) ellipses represent positive and negative correlations, respectively. The numbers in the legend indicate the correlation coefficient.

To confirm the expression patterns of *CmbZIP* genes related to starch synthesis, we used published transcriptome data of all genes from the N11-1 version of the reference genome to determine the fragments per kilobase transcript per million mapped reads (FPKM) (**Table S5**). All *CmbZIP* genes were clustered to four subclades by their expression profiles (**Figure 6A**). The greatest number of *CmbZIP*s were in subclade II, but almost all members (10/19) had very low levels of expression (FPKM < 0.5) in every sample. The 15 members of subclade IV had relatively high expression levels, with an average FPKM ranging from 29.1 to 130.3 (**Table S5**).

Furthermore, we performed a correlation analysis between expression patterns of 59 *CmbZIP*s and four physiological characteristics: total starch content, amylopectin content, amylose content, and starch synthase activity (**Figures 6B**, **S2**; **Table S6**). Fourteen highly expressed *CmbZIP*s, namely *CmbZIP33*, *CmbZIP47*, *CmbZIP14*, *CmbZIP38*, *CmbZIP48*, *CmbZIP56*, *CmbZIP35*, *CmbZIP04*, *CmbZIP39*, *CmbZIP40*, *CmbZIP45*, *CmbZIP06*, *CmbZIP13*, and *CmbZIP07*, were identified to be significantly associated (|r| ≥ 0.67, p < 0.05) with starch accumulation (content of total starch or amylopectin) or activity of starch synthase during chestnut seed development (**Figure 6B**; **Table S6**).

### 2.7 Identification of co-expression networks related to starch accumulation

**Figure 7:**
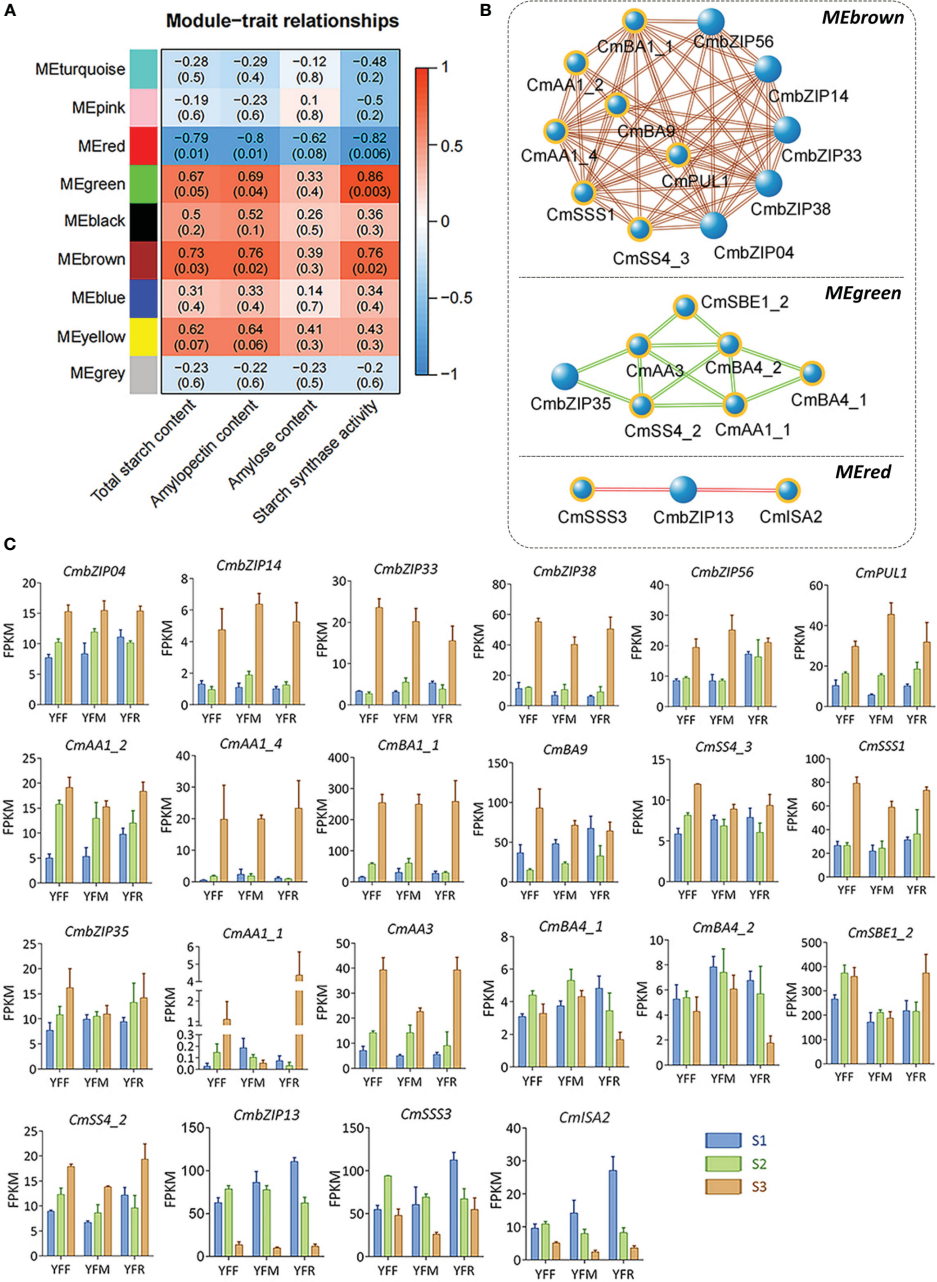
Module–trait relationships, co-expression networks, and module-specific gene expression profiles based on results from the WGCNA. **(A)** The heatmap represents relationships between WNCGA modules and traits. The top and bottom numbers in the heat grid represent the correlation coefficients and p-values (shown in parentheses), respectively. **(B)** The networks represent co-expression relationships of *CmbZIP*s and starch accumulation related genes in three key modules. The blue balls highlighted in indicate starch accumulation related genes; the larger balls indicate *CmbZIP*s. Bold and italic characters indicate the names of key modules. **(C)** The column diagrams describe the expression profiles of genes in panel **(B)** The abbreviations YFF, YFM, YFR, S1, S2, and S3 are the same as those used in **Figure 6**.

To investigate the co-expression networks related to starch accumulation, eight modules with gene numbers ranging from 921 to 4,890 were identified by weighted gene co-expression network analysis (WGCNA) (**Figure S3**; **Table S5**). Analysis of module–trait relationships revealed that three key modules (MEred, MEgreen, and MEbrown) were significantly associated with starch accumulation, with |r| ≥ 0.6 and p < 0.05 (**Figure 7A**). In detail, MEred module was negatively related to contents of total starch (r = -0.79, p = 0.01) and amylopectin (r = -0.8, p = 0.01), and starch synthase activity (r = -0.82, p = 0.006). In contrast, MEbrown module was positively related to contents of total starch (r = 0.73, p = 0.03) and amylopectin (r = 0.76, p = 0.02), and starch synthase activity (r = 0.76, p = 0.02). MEgreen module was also related to amylopectin content (r = 0.69, p = 0.04) and starch synthase activity (r = 0.86, p = 0.003) positively, but not with total starch content. We further identified seven starch accumulation related genes co-expressing with five *CmbZIP*s (*CmbZIP04*, *CmbZIP14*, *CmbZIP33*, *CmbZIP38*, and *CmbZIP56*) in the MEbrown module. Six starch accumulation related genes were found to be co-expressing with one *CmbZIP* (*CmbZIP35*) in the MEgreen module. Two starch accumulation related genes were found to be co-expressing with one *CmbZIP* (*CmbZIP13*) in the MEred module (**Figure 7B**; **Table S7**). The expression profiles of these genes were evaluated by FPKM (**Figure 7C**).

bZIP transcription factors can recognize ACGT sequences in gene promoter regions, particularly A-box (TACGTA), C-box (GACGTC), and G-box (CACGTG) sequences (Izawa et al., 1993). Interestingly, all starch accumulation related genes from key modules contained at least one ACGT sequence in the promoter region, except for *CmPUL1* (**Figure S5**; **Table S7**). A-box occurred at -733 bp and -1,377 bp in promoters of *CmSBE1_2* and *CmAA3*, respectively. C-box was only predicted in the promoter region of *CmBA1_1*, with the positions of -726 bp and -294 bp. G-box was the most frequently identified ACGT sequence in the promoter regions of *CmBA1_1* (-240 bp, -98 bp, and -75 bp), *CmBA4_1* (-289 bp), *CmISA2* (-771 bp), and *CmSSS3* (-648 bp and -595 bp). Based on these findings, we can infer that *CmbZIP*s may regulate the expression of starch accumulation related genes through ACGT cis-elements, and then participate in the accumulation of starch in chestnut seeds.

### 2.8 Identification of interactions of *CmbZIP*s with promoters of starch accumulation related genes

**Figure 8:**
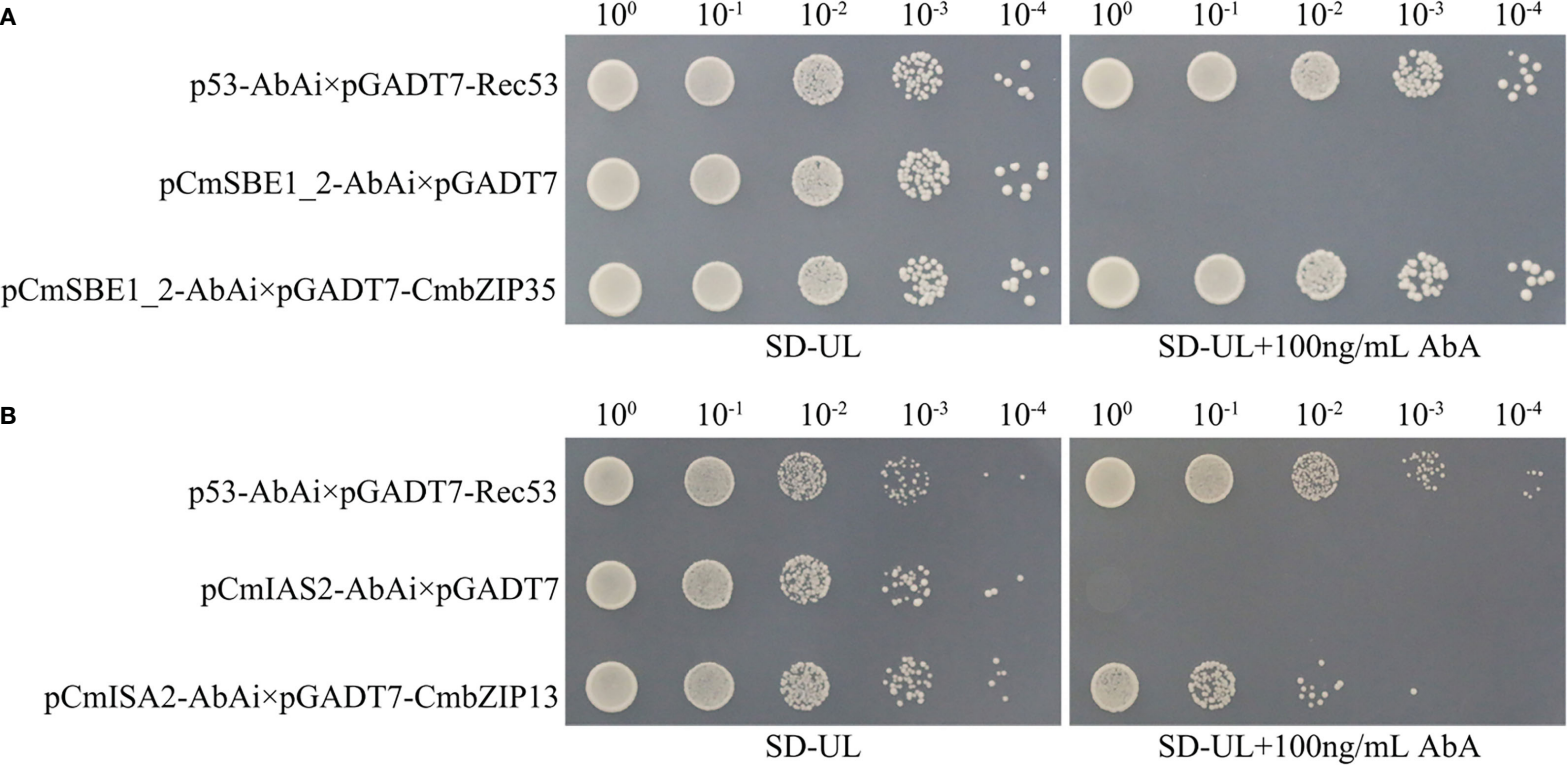
Detection of interactions between *CmbZIP*s and starch accumulation related genes. Yeast one-hybrid assays identified that CmbZIP35 interacted with the promoter of *CmSBE1_2* **(A)**, and CmbZIP13 interacted with the promoter of *CmISA2* **(B)**. The yeast strain containing pCmSBE1_2-AbAi×pGADT7-CmbZIP35 **(A)** or pCmISA2-AbAi×pGADT7-CmbZIP13 **(B)** is the experimental group. The yeast strain containing p53-AbAi×pGADT7-Rec53 is the positive control. The yeast strain containing pCmSBE1_2-AbAi×pGADT7 or pCmISA2-AbAi×pGADT7 is the negative control. All yeast strains were selected on SD-UL+100 ng/mL AbA medium. The numbers (10^0^, 10^-1^, 10^-2^, 10^-3^, and 10^-4^) shown along the top of **(A)** and **(B)** represent the dilution ratios of the yeast solution.

We selected the only two *bZIP* genes in MEgreen and MEred by our interest, *CmbZIP35* and *CmbZIP13*, to identify their potential functions of binding to promoters of starch accumulation related genes. In yeast one-hybrid (Y1H) assays, the Y1H Gold yeast strains containing pCmSBE1_2-AbAi×pGADT7-CmbZIP35 were able to grow on the screening synthetic dropout medium lacking uracil and leucine (SD-UL) containing 100 ng/mL Aureobasidin A (AbA) (**Figure 8A**). Similarly, the Y1H Gold yeast strains containing pCmISA2-AbAi×pGADT7-CmbZIP13 were able to grow under the same conditions (**Figure 8B**). These observations suggested that CmbZIP35 can directly bind to the promoter of *CmSBE1_2*, and CmbZIP13 can bind to the promoter of *CmISA2*.

## 3 Discussion

Previous studies have shown that the bZIP TF family plays an important role in the regulation of plant growth and development as well as resistance to biotic and abiotic stresses (Lee et al., 2006; Schlögl et al., 2008; Song et al., 2020; Wang et al., 2022). Numerous studies have been conducted on bZIP TFs, such as in *Arabidopsis* (Gibalova et al., 2017), wheat (Song et al., 2020), rice (Wang et al., 2013), tobacco (Duan et al., 2022), and apple (An et al., 2018). Although several versions of the Chinese chestnut genome have been published (et al., 2019; Sun et al., 2020; Wang et al., 2020; Hu et al., 2022), to our knowledge, there have been no published studies on the function of bZIP TFs in chestnut. In this study, we identified 59 *bZIP* genes in the chestnut genome, which has a complete genome size of 689.98 Mb (Wang et al., 2020), and further analyzed the characteristics of the *bZIP* genes.

The bZIP domain consists of a basic region and a leucine zipper region (Jakoby et al., 2002). In the present study, we identified the two structural features *via* analyzing conserved motifs, and they were named motif 1 and motif 3 (**Figure S1**); this is similar to the bZIPs in tobacco (Duan et al., 2022). We also identified another 18 motifs in the 59 CmbZIP proteins (**Figure 2B**). Strong clade-specificity and conservation were detected in the motif distribution and phylogenetic analyses of CmbZIPs, and the findings were similar to those reported for pomegranate (Wang et al., 2022), pear (Ma et al., 2021), tobacco (Duan et al., 2022), wheat (Liang et al., 2022), and apple (Li et al., 2016). The bZIPs containing the same motifs might have similar functions (Wang et al., 2022). For example, all members in clade D, which contained motifs 13 and 16, encode the light-inducible protein CPRF2; and most members of clade H, with motifs 5 and 8, may participate in the pathways responding to abiotic stress by encoding ABSCISIC ACID-INSENSITIVE 5-like proteins (**Figure 2B**; **Table S1**). In addition, clade-specificity was observed in the number of introns (**Figure 2C**; **Table S1**), similar to previous studies (Duan et al., 2022; Liang et al., 2022; Wang et al., 2022). These analyses suggested that conserved motifs and gene structure were critical for members in same clade during evolution and functional differentiation (Wang et al., 2022).

WGD drives the evolution and differentiation of plant genome structure (Paterson et al., 2012). WGD, especially segmental and tandem duplications, drive the expansion of gene families (Li et al., 2019; Sun et al., 2019; Wu et al., 2019; Duan et al., 2022). In this study, ten pairs of *CmbZIP*s were identified to have derived from segmental duplication events, and these duplicated genes were clustered into the same clade (**Table 1**). Based on the Ka/Ks ratio, we found that *CmbZIP* genes have undergone purifying and/or positive selection events. It is likely that seven pairs of *CmbZIP*s have undergone purifying selection pressures before the divergence of *C. mollisima* and *Quercus robur* (18.3 Mya) (Wang et al., 2020). These genes may have maintained similar functions. *CmbZIP09*/*45* and *CmbZIP19*/*25* might have undergone positive selection after the formation of the genome of *C. mollisima*, which might play an important role in promoting the evolution of *C. mollisima*.

In the phylogenetic analysis, CmbZIP proteins were grouped according to their homology with AtbZIPs (Jakoby et al., 2002). The bZIPs with highly similar protein sequences were clustered into the same clade, in which the members maintained similar functions. Previous studies have shown that two G-box binding factor (GBF) encoding genes *AtbZIP41* (*GBF1*) and *AtbZIP55* (*GBF3*) might play roles during seed maturation (Chern et al., 1996; Jakoby et al., 2002). Therefore, it is possible that CmbZIPs in clade B, especially CmbZIP33, CmbZIP12, and CmbZIP07 (**Figure 1**), may be involved in the maturation of the chestnut kernel. Furthermore, AtbZIPs in clades F, H, and L might be involved in abiotic and biotic stress response (Jakoby et al., 2002; Liu et al., 2010; Skubacz et al., 2016; Lapham et al., 2018). CmbZIP16/17/58 and CmbZIP41 were, respectively, closely related to OsbZIP33/58 and TabZIP28/TubZIP28, which have been shown to be involved in starch synthesis (Cai et al., 2002; Song et al., 2020). Therefore, we hypothesize that CmbZIP16/17/58 and CmbZIP41 may participate in the regulation of starch synthesis.

In starchy seeds, such as rice and wheat, starch acts as a sink of carbon allocation. In developing seeds, starch is synthesized from sucrose, which is catalyzed by enzymes and regulated by TFs (MacNeill et al., 2017). Previous studies found that OsbZIP58 participates in the regulation of starch synthesis in rice by binding to the promoters of *OsAGPL3*, *OsGBSS*, *OsSSIIa*, *OsSBE1*, *OsBEIIb*, and *OsISA2*, which encode enzymes critical to the starch synthesis process (Wang et al., 2013). Similarly, a previous study reported that TubZIP28 and TabZIP28 are both capable of binding to the promoter of cytosolic AGPase encoding gene to enhance the total starch content (Song et al., 2020). In our study, the expression pattern of *CmbZIP* genes in developing chestnut seeds was further analyzed (**Figure 6**). The results showed that most *bZIP* genes were highly expressed 70–94 DAP, except for *CmbZIP08*, *CmbZIP18*, *CmbZIP24*, *CmbZIP28*, *CmbZIP29*, *CmbZIP34*, *CmbZIP36*, *CmbZIP49*, *CmbZIP50*, and *CmbZIP59*, which showed overall low levels of expression. This suggested that *CmbZIP* genes might participate in the regulation of chestnut seed maturation. In the correlation and co-expression analysis, we identified that the expression of seven *bZIP*s from the modules containing *CmbZIP04*, *CmbZIP13*, *CmbZIP14*, *CmbZIP33*, *CmbZIP35*, *CmbZIP38*, and *CmbZIP56* was closely related to starch (especially amylopectin) accumulation in chestnut seeds (**Figure 7**; **Table S6**) (Li et al., 2021). In the analysis of cis-elements, several ‘ACGT’ elements, including A- and G-boxes, were found in the promoter regions of *CmISA2* (encoding an isoamylase-type starch de-branching enzyme) and *CmSBE1_2* (encoding a starch branching enzyme) (**Figure S5**). *CmISA2* and *CmSBE1_2* can modify glucan. In the Y1H assays, CmbZIP13 and CmbZIP35 TFs were found to directly bind to the promoters of *CmISA2* and *CmSBE1_2*, respectively (**Figure 8**). Therefore, we hypothesize that *CmbZIP13* and *CmbZIP35* genes might participate in starch accumulation in the chestnut seed by interacting with *CmISA2* and *CmSBE1_2*, respectively.

## 4 Materials and methods

### 4.1 Identification of *CmbZIP* genes

The protein sequence data from the previously published genome of the N11-1 Chinese chestnut, a seedling chestnut cultivar, were obtained from the Genome Warehouse in BIG Data Center under accession number GWHANWH00000000 (https://bigd.big.ac.cn/gwh) (Wang et al., 2020). A hidden Markov model (HMM) was used to identify chestnut bZIP candidates, and the HMM profile of bZIP (PF00170) was downloaded from the Pfam protein database (http://pfam.xfam.org/) (Finn et al., 2016). To identify *CmbZIP* genes, the hmmsearch tool of HMMER 3.0 software (Potter et al., 2018) was used to retrieve a domain similar to the bZIP domain in chestnut. The hmmbuild tool was used to rebuild the new HMM profile to re-identify CmbZIP protein sequences. Finally, these protein sequences were confirmed as bZIPs *via* the conserved domain using Pfam (http://pfam.xfam.org/) and Batch CD-Search web tool (https://www.ncbi.nlm.nih.gov/cdd/) (Lu et al., 2020).

### 4.2 Sequence and phylogenetic analyses

All CDS sequences of *CmbZIP* genes were submitted to ExPASy (https://www.expasy.org) to determine gene length, amino acid length, relative molecular weight, isoelectric point, hydrophilicity, stability, and other physicochemical properties analysis. The protein sequences of 59 *CmbZIP* genes, 23 *AtbZIP* genes (Jakoby et al., 2002), *OsbZIP20* (Izawa et al., 1994), *OsbZIP33* (Cai et al., 2002), *OsbZIP58* (Wang et al., 2013), *TubZIP28*, and *TabZIP28* (Song et al., 2020) were imported into MEGA X, and ClustalW was used for multiple sequence alignments (Kumar et al., 2018). A Neighbor-Joining (NJ) phylogenetic tree was constructed using MEGA X software with bootstrapping set to 1,000. The optional parameters substitution model and gaps data treatment were set to p-distance and partial deletion, respectively. EvolView (https://www.evolgenius.info/evolview/) was used to annotate and visualize the phylogenic trees (Subramanian et al., 2019). CmbZIP proteins were grouped according to their homology with AtbZIPs (Jakoby et al., 2002).

### 4.3 Gene structure and conserved motif analyses

A gene structure analysis of 59 *CmbZIP* genes was performed with general feature format (GFF) file, and visualized using the online software Gene Structure Display Server (http://gsds.cbi.pku.edu.cn/) (Hu et al., 2015). Conserved motifs were identified using Multiple Expectation Maximization for Motif Elicitation (MEME version 5.1.0) (Bailey et al., 2015) with motif width set to 8–100 and the parameter of maximum motif number set to 20.

### 4.4 Chromosomal localization and synteny analyses

All *CmbZIP* genes were mapped to Chinese chestnut chromosomes based on physical location information from the GFF file using Mapchart 2.32 software (Voorrips, 2002). Multiple Collinearity Scan toolkit (MCScanX) was used to identify the syntenic gene pairs within the genome, using the results from all-*vs*-all BLASTP analysis (Wang et al., 2012). Results were displayed with Circos (version 0.69-8) software (Krzywinski et al., 2009). The values of Ka and Ks were calculated using the KaKs-calculator 2.0 (Wang et al., 2010). The divergence-times of duplicated gene pairs were estimated using the Ks value with the formula *T* = *Ks*/*2r*, where *T* is the divergence-time and *r* is the divergence rate of nuclear genes from plants (*r* = 1.5 × 10^-8^) (Koch et al., 2000; Huang et al., 2016). We used the Python version of MCscan to analyze the synteny between the genomes of *C. mollissima*, *A. thaliana*, *O. sativa*, *T. aestivum*, and *M. domestica* (Tang et al., 2014).

### 4.5 Expression profile and co-expression analyses based on RNA-seq

The published RNA-seq data were obtained from the sequence read archive (SRA) in NCBI (accession number PRJNA540079) (Li et al., 2021). All seed samples were collected at 70, 82, and 94 days after pollination from three crosses: ‘Yongfeng 1’×’Yongfeng 1,’ ‘Yongfeng 1’×’Yimen 1,’ and ‘Yongfeng 1’×’Yongren Zao’ (Li et al., 2021). RNA-seq read-files were converted from SRA to fastq format using sratoolkit3.0 (Goldberg et al., 2009). The mapping genome used in the RNA-seq analysis was changed from the previous version of the genome assembly to N11-1. The expression profiles of *CmbZIP*s were determined using Tophat2 software (Kim et al., 2013; Wang et al., 2020; Li et al., 2021). The FPKM value was used to evaluate the expression level of each gene. The correlation coefficients and p values between the log_10_FPKM of *CmbZIP*s and four physiological characteristics (i.e., total starch content, amylopectin content, amylose content, and starch synthase activity) published in a previous study were estimated using GraphPad Prism version 6.02 for Windows (Li et al., 2021; https://www.graphpad.com/). The co-expression modules were identified using WGCNA with the R package (Langfelder and Horvath, 2008). The co-expression networks were generated using Cytoscape software (Otasek et al., 2019).

### 4.6 Identification of cis-elements in gene promoter regions

We retrieved sequences 1,500 bp upstream of the transcription start site of starch accumulation related genes. These sequences were submitted to PlantCARE to identify cis-elements, which might affect gene expression and function (Rombauts et al., 1999).

### 4.7 Sequence cloning and yeast one-hybrid assays

The open reading frames of *CmbZIP35* and *CmbZIP13* were cloned by reverse transcription PCR using RNA from developing seeds of *C. mollissima* cultivar ‘Yanbao.’ We confirmed these sequences using DNAMAN 6.0 software. Subsequently, we fused the two sequences into the pGADT7 vector to construct the pGADT7-CmbZIP35 and pGADT7-CmbZIP13 recombinant plasmids, respectively. The *CmSBE1_2* and *CmISA2* promoter fragments were cloned by PCR using DNA from ‘Yanbao’ seeds. The fragments were confirmed and inserted into the pAbAi vector to construct the pCmSBE1_2-AbAi and pCmISA2-AbAi recombinant plasmids. All primer sequences are listed in **Table S8**.

To determine the optimal AbA concentration, the Y1H Gold yeast strain containing the recombinant pAbAi plasmids were grown on SD-UL screening medium supplemented with different AbA concentrations (Yang et al., 2019). Then, Y1H Gold yeast cells were co-transformed with pGADT7-CmbZIP35 and pCmSBE1_2-AbAi plasmids, as well as pGADT7-CmbZIP13 and pCmISA2-AbAi plasmids. Interactions were detected on SD-UL selection medium that was supplemented with 100 ng/mL AbA.

## 5 Conclusions

A total of 59 *CmbZIP* genes were identified in Chinese chestnut. These *CmbZIP*s were clustered into 13 clades with clade-specific motifs and structures. Segmental duplication was determined as the major driving force of the expansion of the *CmbZIP* gene family. *CmbZIP04*, *CmbZIP13*, *CmbZIP14*, *CmbZIP33*, *CmbZIP35*, *CmbZIP38*, and *CmbZIP56* were confirmed to be highly correlated with starch accumulation in chestnut seeds. We demonstrated that *CmbZIP13* and *CmbZIP35* may regulate starch accumulation in the chestnut seed by binding to the promoters of *CmISA2* and *CmSBE1_2*, respectively. These results indicated that *CmbZIP* genes contained information related to starch accumulation in chestnut seeds, which can be used in future functional analysis and breeding studies.

## Data availability statement

The datasets presented in this study can be found in online repositories. The names of the repository/repositories and accession number(s) can be found in the article/**Supplementary Material**.

## Author contributions

XW and HZ designed the experiments. MW and YL collected data of Chinese chestnut genome and RNA-seq. NJ and JL drew all figures of this study. PZ and JL carried out the yeast one-hybrid experiments. PZ and XW performed the bioinformatic analysis and wrote the paper. DW and JZ directed and revised the manuscript. All authors contributed to the article and approved the submitted version.
