# Supplementary material for: Genome-wide identification and characterization of the bZIP gene family and their function in starch accumulation in Chinese chestnut (Castanea mollissima Blume)
Source: Front Plant Sci. 2023 Apr 3;14:1166717. doi: 10.3389/fpls.2023.1166717 (PMC10106562; doi:10.3389/fpls.2023.1166717)
Supplement: Supplementary file 1 [file DataSheet_1.pdf]

Table S2. Information of 20 conservative motifs of 59 CmbZIP proteins.

Motif	Length (aa)	Logo of motif
1	23	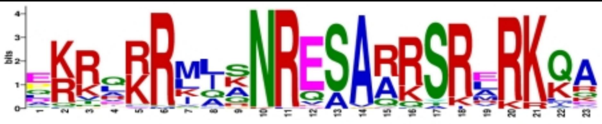
2	100	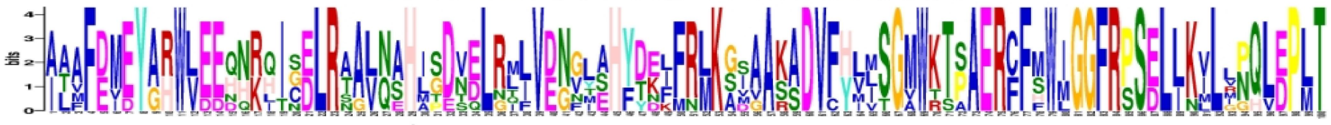
3	21	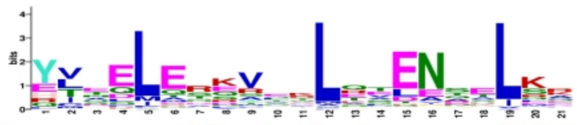
4	41	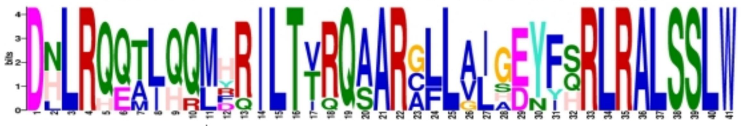
5	15	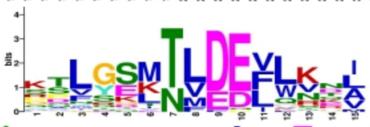
6	32	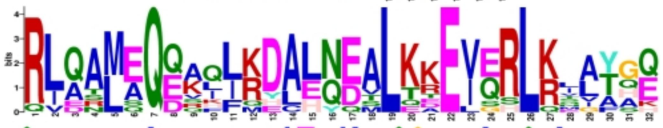
7	39	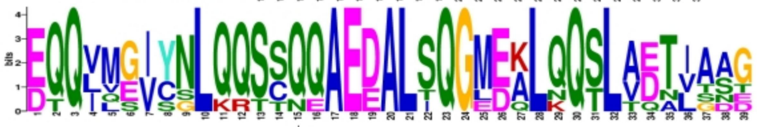
8	8	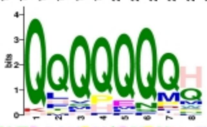
9	100	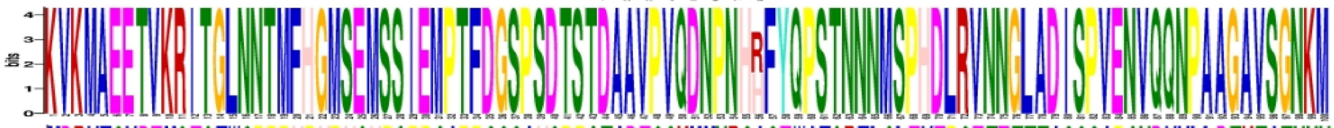
10	100	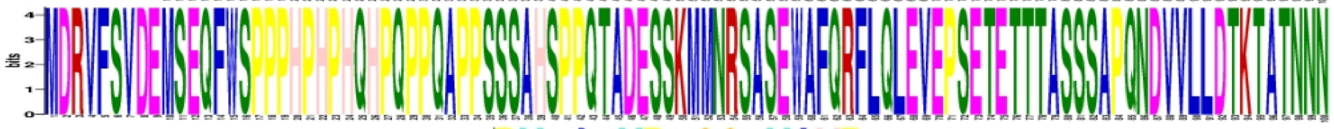
11	15	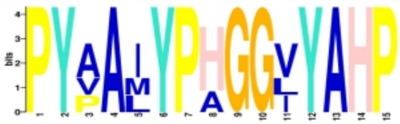
12	100	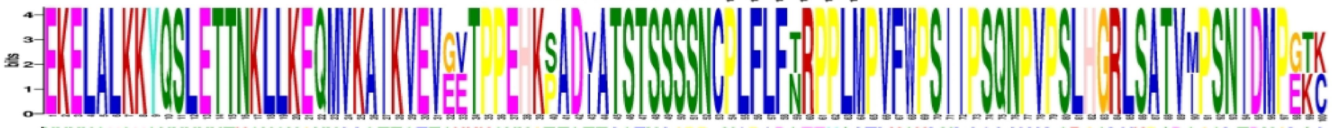
13	100	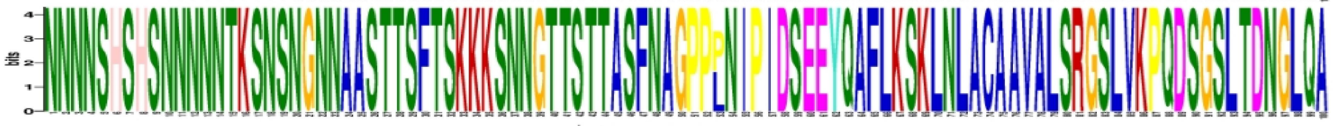
14	8	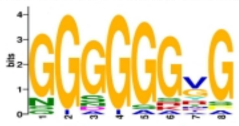
15	14	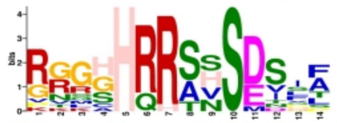
16	56	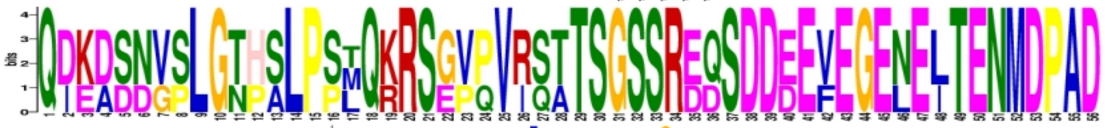
17	19	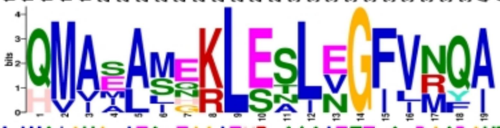
18	98	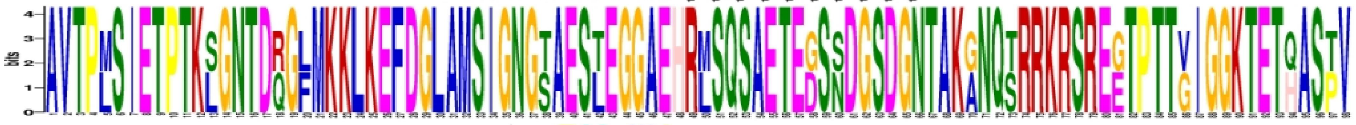
19	15	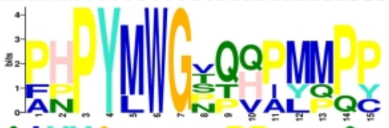
20	20	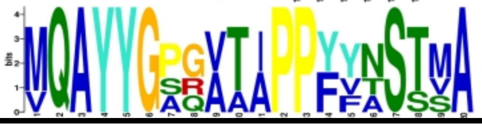
