# Supplementary material for: Genome-wide identification and characterization of the bZIP gene family and their function in starch accumulation in Chinese chestnut (Castanea mollissima Blume)
Source: Front Plant Sci. 2023 Apr 3;14:1166717. doi: 10.3389/fpls.2023.1166717 (PMC10106562; doi:10.3389/fpls.2023.1166717)
Supplement: Supplementary file 2 [file DataSheet_2.pdf]

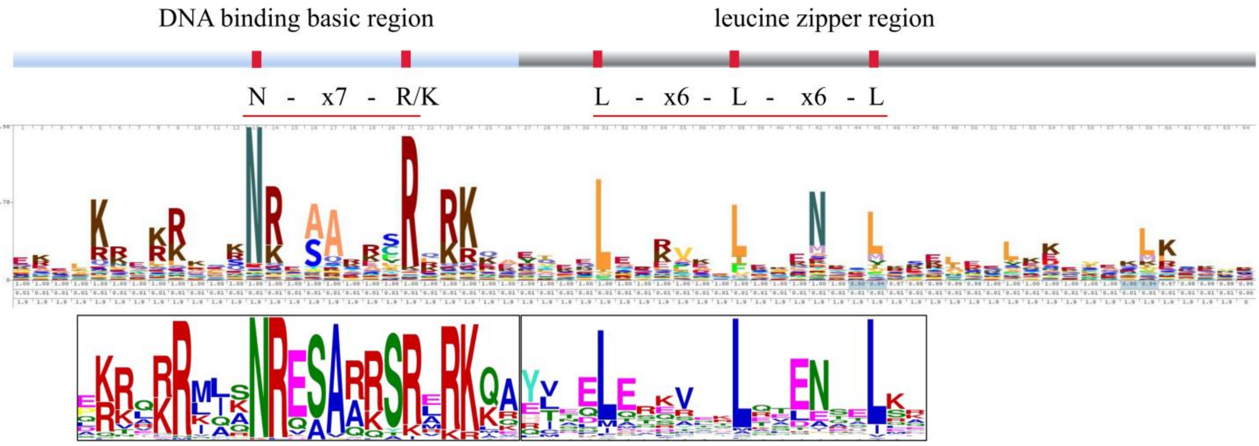


**Figure S1.** Logo of bZIP domain comparsing with motif 1 and motif 3.

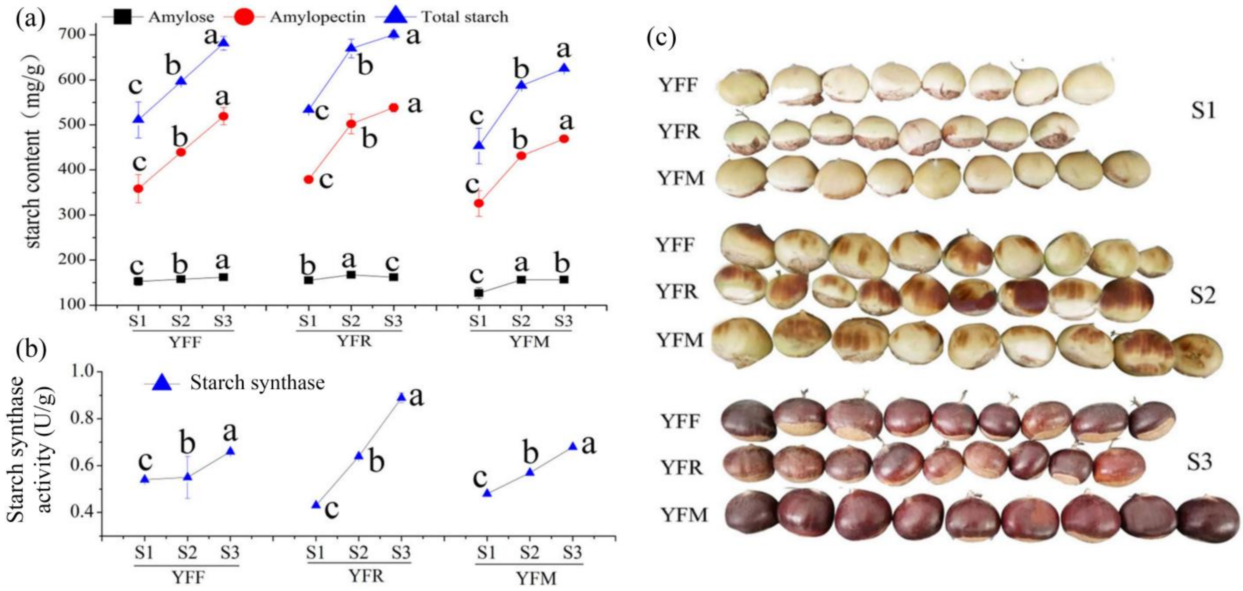


**Figure S2.** Physiological characteristics of chestnut samples modified from previous study (Li et al., 2021). (a) Contents of starch in chestnut seeds. (b) Activity of starch synthase in chestnut seeds. (c) Photo of chestnut seeds at three stages. Note: The difference significance analysis is at 0.05 level. YFF, YFM and YFR indicate seeds from crosses of ‘Yongfeng 1’×‘Yongfeng 1’, ‘Yongfeng 1’×‘Yimen 1’ and ‘Yongfeng 1’×‘Yongren Zao’, respectively. S1, S2 and S3 indeicate 70, 82 and 94 days after pollination respectively.

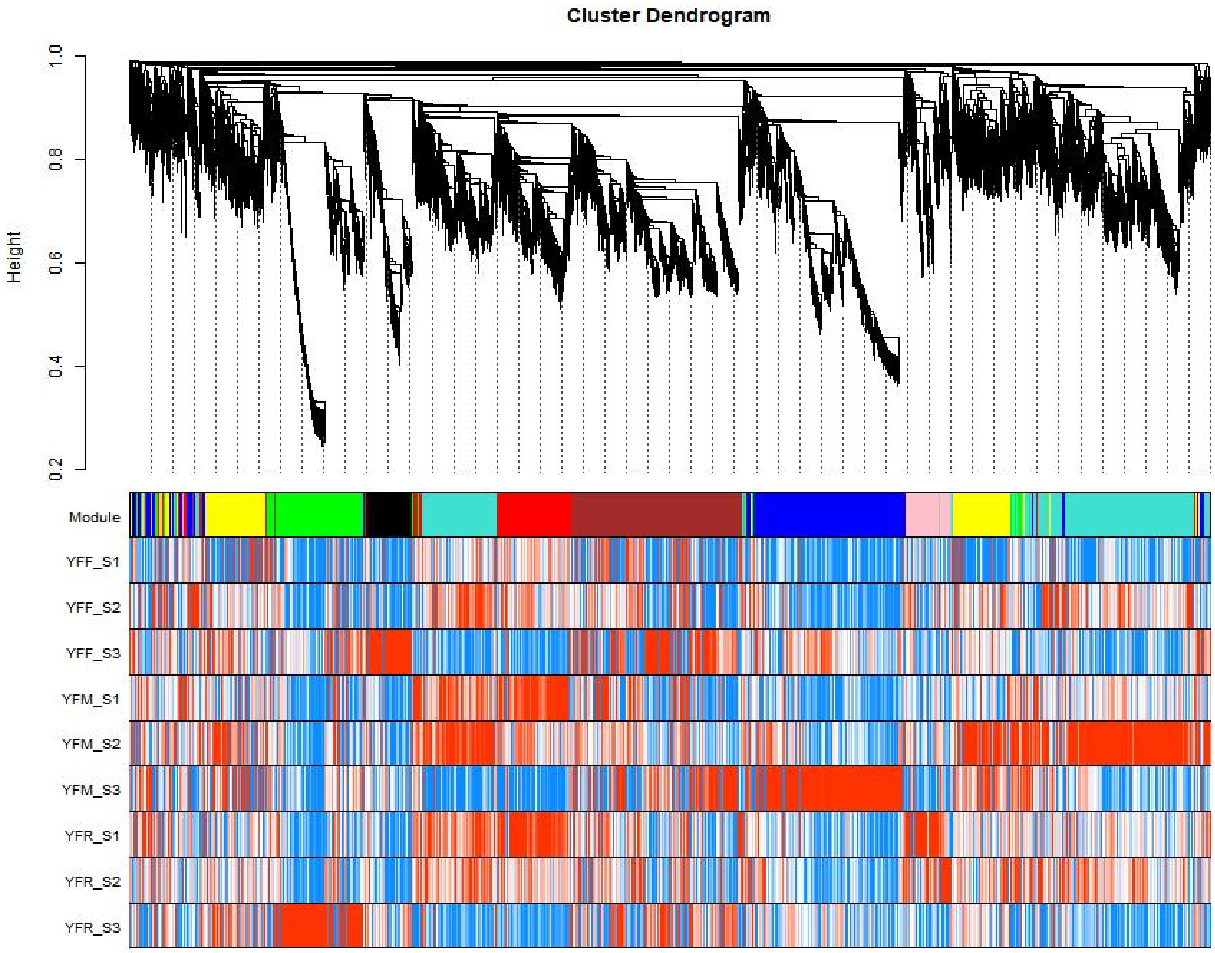


**Figure S3.** The upper clustering dendrogram shows to topological relationship of expressed genes, and the lower panel shows modules in designated colors and expression patterns in each sample (red: high expressed, blue: low expressed).

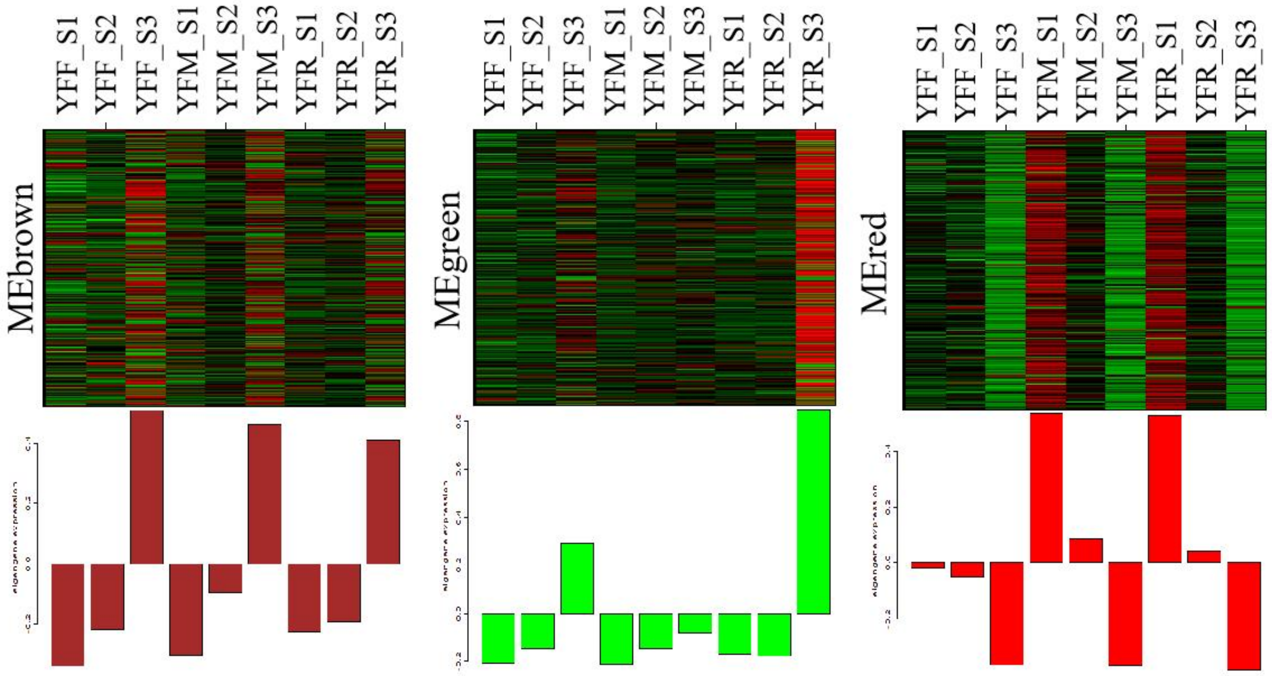


**Figure S4.** The eigengene expression patterns of three key modules.

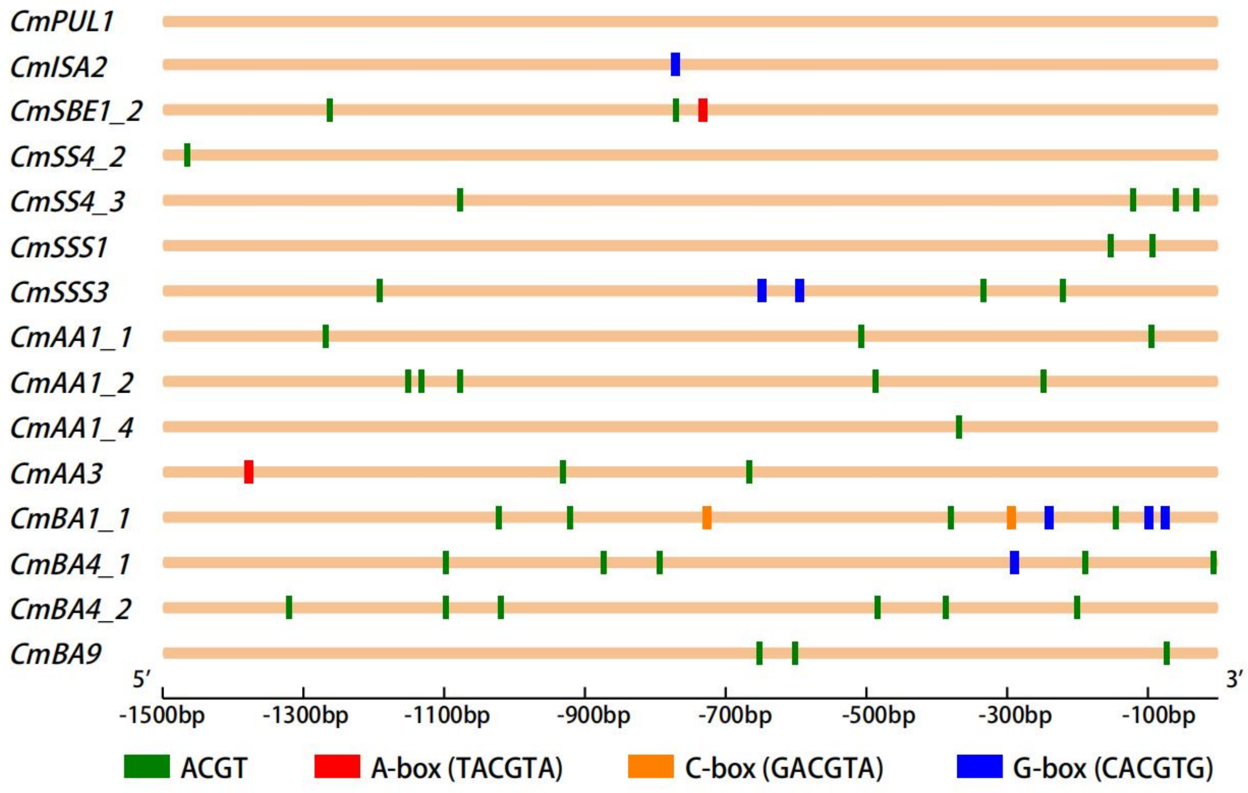


**Figure S5.** The distribution of cis-elements containing ACGT sequence in the promoter region of starch metabolism related genes, which were identified in MEbrown, MEgreen and MEred modules.
